# Successful treatment of descending necrotizing mediastinitis by combining a cervical approach with artificial pneumomediastinum and video-assisted thoracoscopic surgery: a case report

**DOI:** 10.1186/s44215-023-00094-7

**Published:** 2023-08-02

**Authors:** Mitsuhiro Tsuboi, Daisuke Matsumoto, Toshiyuki Hirose

**Affiliations:** Department of Surgery, Tokushima Prefectural Central Hospital, Tokushima, Japan

**Keywords:** Descending necrotizing mediastinitis, Mediastinoscopy, Artificial pneumomediastinum, Video-assisted thoracoscopic surgery

## Abstract

**Background:**

Descending necrotizing mediastinitis (DNM) is a fatal disease that originates from odontogenic, pharyngeal, and cervical infections and spreads to the mediastinum. Although adequate drainage of the neck and mediastinum is important, it may be excessively invasive. We report a case of DNM treated using a cervical approach with artificial pneumomediastinum and video-assisted thoracoscopic surgery (VATS).

**Case presentation:**

A 69-year-old male patient who underwent gastrointestinal endoscopy 4 days prior to presentation was referred to our hospital for mediastinal air observed using computed tomography. He was diagnosed with DNM, and an emergency surgery was performed. We created a collar incision and performed drainage and debridement of the cervix. Then, we performed drainage of the retropharyngeal and upper mediastinum by inserting the mediastinoscope through the collar incision and creating artificial pneumomediastinum. Next, drainage of the thoracic cavity was performed during VATS. Subsequently, the abscess resolved, and the patient was discharged on postoperative day 28 without severe complications.

**Conclusions:**

Surgical drainage using a cervical approach with artificial pneumomediastinum and VATS was useful for treating this case of DNM. This procedure could be a viable option for minimally invasive surgery for DNM.

## Background

Descending necrotizing mediastinitis (DNM) is a fatal disease that originates from odontogenic, pharyngeal, and cervical infections and spreads downward to the mediastinum [1]. Furthermore, DNM involves the mediastinal connective tissue, and it has a mortality rate ranging from 25 to 40% [2]. Both adequate drainage of the neck and mediastinum and intravenous broad-spectrum antibiotics are necessary to treat DNM, and therefore, several surgical approaches have been reported. Here, we report a case of DNM treated using a cervical approach with artificial pneumomediastinum and video-assisted thoracoscopic surgery.

## Case presentation

A 69-year-old male patient underwent gastrointestinal endoscopy because of stomach discomfort at a nearby medical clinic. Intubation was difficult and required several attempts. The fiberoptic gastroscope showed a fundic gland polyp. There was no obvious evidence of perforation. Soon after the endoscopy, the patient reported neck pain on the left side; therefore, he was administered antibiotics. At 4 days after the endoscopy, computed tomography (CT) showed mediastinal air; therefore, the patient was referred to our hospital.

**Fig. 1:**
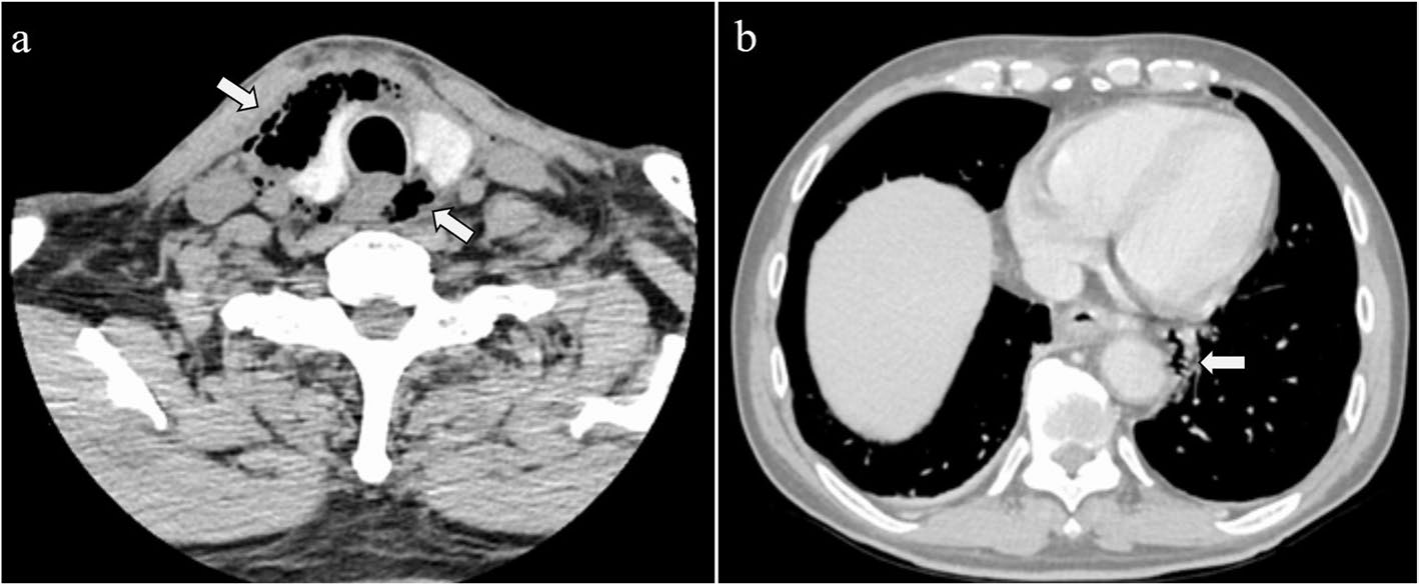
Cervicothoracic computed tomography (CT) image showing gas bubbles in the cervical spaces (**a**) and posterior mediastinum extending toward the esophagogastric junction (**b**). Arrows in **a** and **b** indicate gas bubbles

**Fig. 2:**
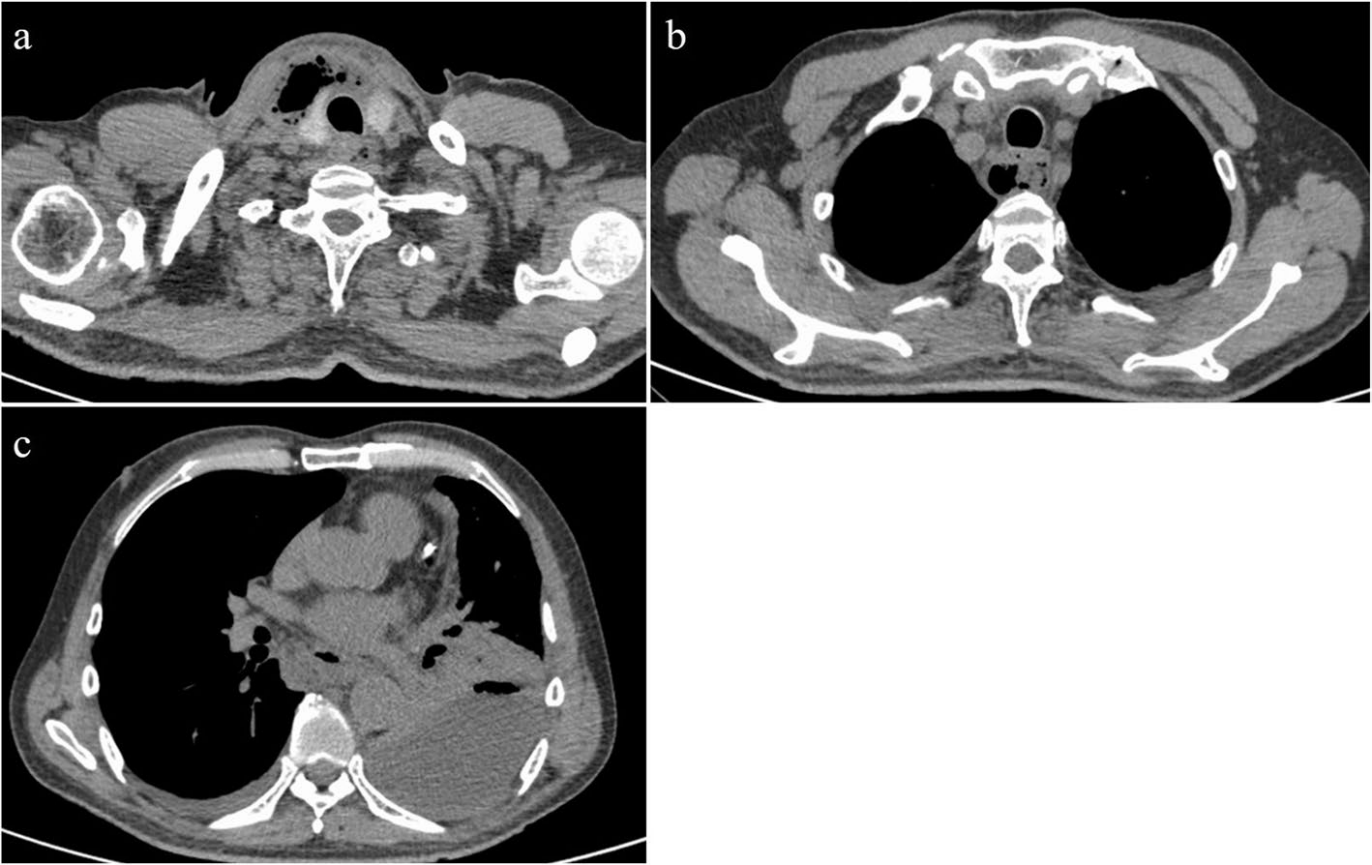
Chest computed tomography (CT) image at 3 days after admission showing gas bubbles and a pool of pus in the cervix (**a**), gas bubbles in the superior mediastinum (**b**), and left thoracic effusion (**c**)

At the time of presentation to the previous physician, the patient had a fever of 38.0 °C. Furthermore, he had used antipyretic medication prior to presenting to our hospital. On admission to our hospital, the patient’s vital signs were as follows: temperature, 36.2 ℃: pulse rate, 92 bpm; blood pressure, 137⁄75 mmHg; respiration rate, 28 breaths/min; and SpO_2_, 100% on room air. His skin from the front of the neck to the anterior chest was swollen and red. He had a medical history of diabetes, hypertension, dyslipidemia, atrial fibrillation, and myocardial infarction. He was using oral hypoglycemic drugs, antihypertensive drugs, and aspirin as an anticoagulant. The laboratory test results were as follows: white blood cell (WBC) count, 10,600/mm^3^; C-reactive protein (CRP) level, 28.9 mg/dL; hemoglobin A1c, 6.4%; and blood sugar, 193 mg/dL. A cervicothoracic CT image demonstrated gas bubbles (surgical emphysema) in the cervical spaces and posterior mediastinum that extended down toward the esophagogastric junction (Fig. 1). After incision and drainage of the neck under local anesthesia in the emergency department, a small amount of purulent discharge from the neck was observed, which was then irrigated with saline. The patient was hospitalized and administered tazobactam/piperacillin. Although the pharyngeal fibers observed by the previous physician did not exhibit any obvious perforation, we speculated that the infection was caused by a microperforation of the pharynx or esophagus after endoscopy. Therefore, we restricted his oral intake preoperatively. At 3 days after admission, the redness and swelling of the neck skin disappeared; however, the blood test results indicated a WBC count of 15,200/mm^3^, and a chest CT image demonstrated a small fluid collection in the posterior mediastinum and left thoracic effusion (Fig. 2). The patient was diagnosed with DNM, and emergency surgery was performed on the same day.

**Fig. 3:**
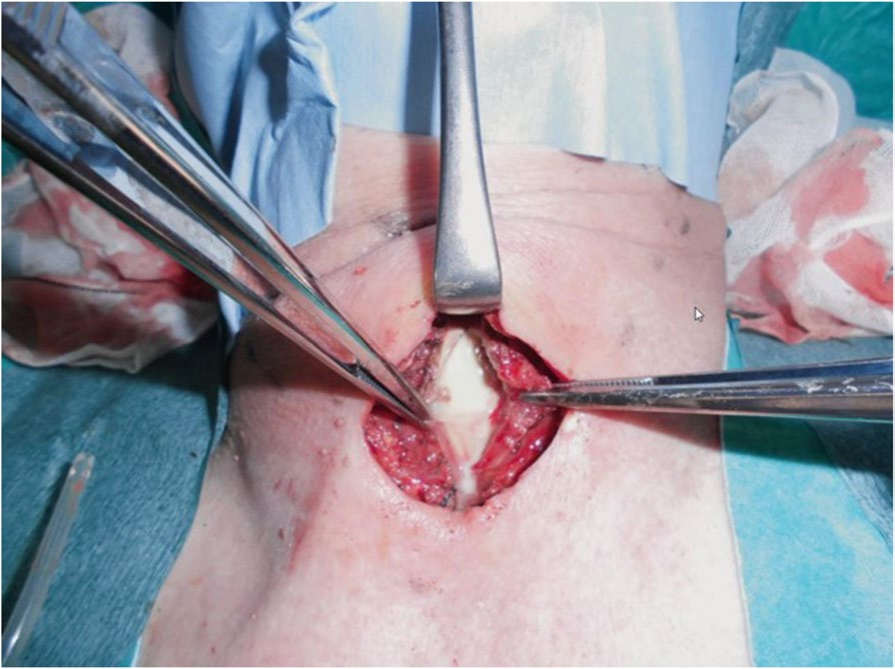
Intraoperative findings. Yellowish-white purulent fluid under the cervical neck muscles

**Fig. 4:**
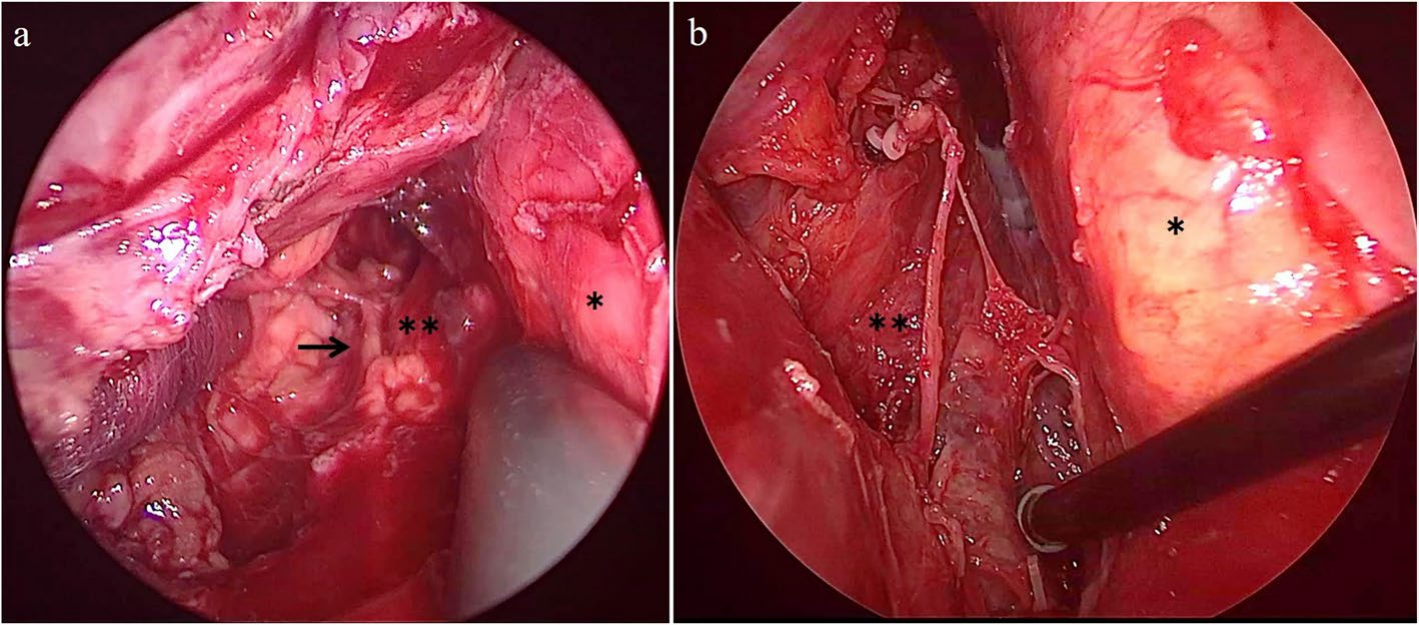
**a** Mediastinoscopic findings. The open abscess cavity on the left lateral side of the esophagus in the cervicothoracic transition area is shown. Arrow indicates the left inferior laryngeal nerve. The trachea (*) and esophagus (**) are shown. **b** Thoracoscopic findings. The open abscess cavity between the esophagus and descending aorta is shown. The descending aorta (*) and posterior wall of the esophagus (**) are shown

Under general anesthesia, the patient was placed in the supine position, with the neck slightly extended. Intubation was performed using a single-lumen tube. A collar incision was created, and the anterior cervical muscles were divided. A large amount of yellowish-white purulent fluid was detected under the cervical neck muscles (Fig. 3). This suspicious fluid was removed completely, and the abscess cavity was irrigated thoroughly. Then, the thyroid and trachea were exposed. The left inferior laryngeal nerve was identified and exposed completely (Fig. 4a). A wound protector was inserted in the cervical wound and attached to the wound retractor (Alnote-LAPSINGLE; Alfresa, Osaka, Japan), which comprised a unit with a cover to prevent gas leakage, three 10-mm trocars, and one 12-mm trocar.

**Fig. 5:**
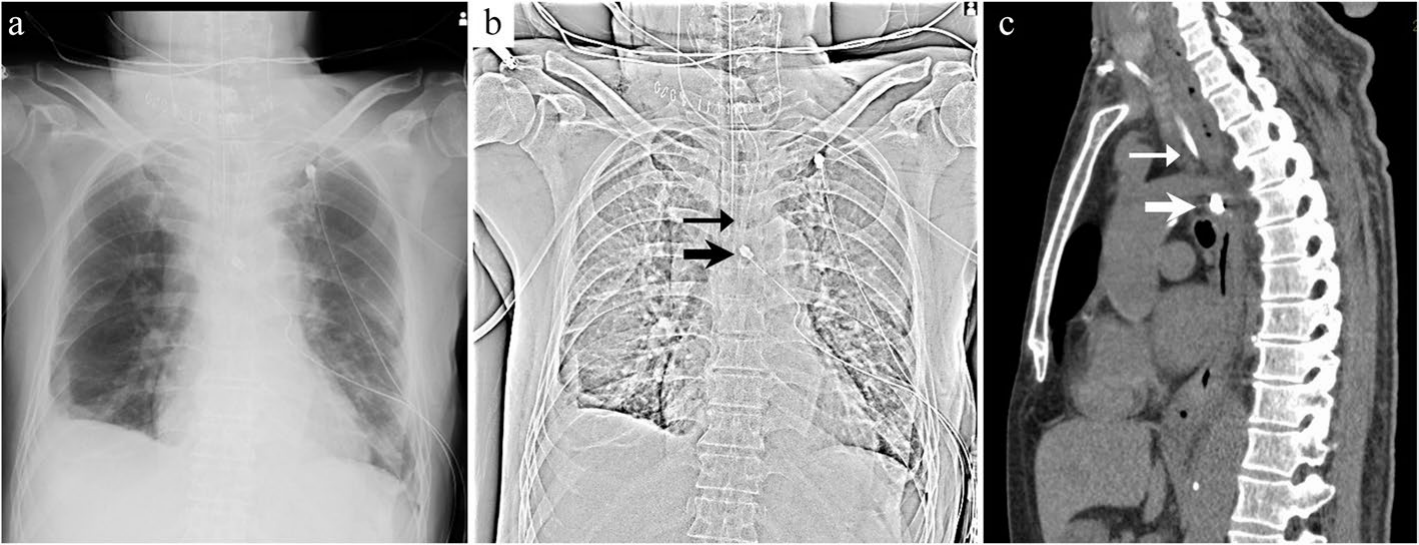
**a**, **b** Postoperative chest radiography image. **c** Postoperative chest computed tomography (CT) image. The tips of the mediastinal drain and chest drain are in close proximity. The thin arrow indicates the tips of the mediastinal drain. The thick arrow indicates the tips of the drain on the dorsal side of the esophagus through the chest cavity

After a 5-mm flexible scope was inserted, pneumomediastinum was created using carbon dioxide insufflation (8 mmHg). Dissection was performed inferiorly along the left wall of the trachea using a cherry dissector (BCD 10; Ethicon Endo-Surgery Inc., Cincinnati, OH). After dissecting the abscess cavity as caudally as possible, 5-mm drainage tubes were placed bilaterally in the neck spaces and superior mediastinum. Subsequently, VATS comprising single-lung ventilation with an endobronchial blocker was initiated with the patient in the right lateral position. A 1-cm port and two 3-cm ports were placed. Turbid pleural effusion was detected in the left thoracic cavity. After approaching the posterior mediastinum and opening the mediastinal pleura, yellow purulent discharge was detected. The abscess cavity was dissected between the esophagus and descending aorta, and the purulent fluid and necrotic tissue in the thoracic cavity and posterior mediastinal space were removed (Fig. 4b). After intrathoracic irrigation, two 20-Fr chest tubes were placed in the thoracic cavity and on the dorsal side of the esophagus, and the wounds were closed. The total operative time was 395 min. Although artificial pneumomediastinum was created, the postoperative imaging showed no obvious subcutaneous or mediastinal emphysema (Fig. 5a) and demonstrated that the tips of the upper mediastinal drain inserted from the neck and posterior mediastinal drain through the thoracic cavity were found in close proximity (Fig. 5b, c). This may have contributed to adequate drainage. After surgery, the patient was admitted to the intensive care unit without intubation.

Culture results of the abscess revealed infection with *Streptococcus anginosus*; therefore, an intravenous antibiotic (meropenem 2 g/day) was administered. At 1 week after surgery, there was no sign of leakage in the gastrointestinal tract, and the patient was allowed a diet per os. After removal of the drains, the patient was discharged on postoperative day 28 without severe complications.

## Discussion and conclusions

DNM originates from odontogenic, pharyngeal, and cervical infections and spreads to the mediastinum via the pretracheal, perivascular, and retrovisceral (prevertebral) spaces [3, 4]. Several surgical procedures have been reported for DNM, including a transcervical approach, subxiphoid approach, median sternotomy, thoracotomy, and thoracoscopic approach; however, the most important aspect of treatment is adequate drainage of the abscess cavity and progression route. Estrera reported that a delayed diagnosis and inadequate drainage are the primary underlying factors contributing to the high mortality rates associated with DNM [1]. Marty-Ane reported that surgical drainage using cervical and thoracic approaches (including thoracotomy) can improve survival [3]. Additionally, mortality rates associated with DNM are significantly higher for patients who underwent cervical drainage alone than for those who underwent cervical drainage and mediastinal drainage [4]. However, some reports have indicated that VATS is a less invasive and more effective modality for DNM [5–7]. VATS is sometimes difficult because of limited visual field and working space, especially in the cervicothoracic transition area [8]; therefore, we speculate that drainage of this area could be inadequate. Thoracotomy [3], median sternotomy [9], and the clamshell approach [10] are radical surgical treatments for DNM; however, these procedures can be excessively invasive when performed in patients with debilitation caused by DNM.

We performed mediastinoscopic surgery through a collar incision with artificial pneumomediastinum for this case. To the best of our knowledge, this is the first report of a DNM treated using this method. Mediastinoscopic surgery with artificial pneumomediastinum has been used for esophagectomy [11] and mediastinal parathyroidectomy [8]; however, it has several advantages. First, pneumomediastinum makes it possible to achieve adequate drainage without sternotomy and thoracotomy, especially in the upper mediastinum, because the air pressure allows for visualization of the surgical field. Additionally, the inferior laryngeal nerve can be easily identified visually during the cervical approach at the beginning of surgery; therefore, an injury can be avoided. Second, the loose connective tissue can be dissected easily because of the air pressure created by pneumomediastinum. Third, this method allows sequential drainage along the route of the descending infection, from the neck to the thoracic cavity, through the mediastinum. Studies have reported that the surgical approach should depend on the extent of infection spread [12]. Overall, we believe this method can contribute to adequate drainage and prevent infection recurrence.

In conclusion, surgical drainage achieved by the use of a cervical approach with artificial pneumomediastinum and VATS was successfully used for treating this case of DNM. Therefore, this minimally invasive surgical option should be considered for DNM cases in the future.

## Data Availability

Not applicable.

## Abbreviations

CRP: C-reactive protein
CT: Computed tomography
DNM: Descending necrotizing mediastinitis
VATS: Video-assisted thoracoscopic surgery
